# Preterm Birth and Risk of Psychiatric Disorders: A Register-Linkage Cohort Study: Liens entre la naissance prématurée et le risque de troubles psychiatriques : une étude de cohorte avec couplage de registres

**DOI:** 10.1177/07067437251389872

**Published:** 2025-10-28

**Authors:** Jude Balit, Ophélie Collet, Seungmi Yang, Sylvana M. Côté, Anne Monique Nuyt, Thuy Mai Luu, Massimiliano Orri

**Affiliations:** 1 Department of Epidemiology, Biostatistics, and Occupational Health, 5620 McGill University, Montreal, Quebec, Canada; 2 McGill Group for Suicide Studies, Douglas Mental Health Research Institute, Department of Psychiatry, 5620 McGill University, Montreal, Quebec, Canada; 3 School of Public Health, 5622 Université de Montréal, Montreal, Quebec, Canada; 4 Azrieli Research Center, 25461 Centre Hospitalier Universitaire Sainte-Justine, Montreal, Quebec, Canada; 5 Department of Pediatrics, Faculty of Medicine, 5622 Université de Montréal, Montreal, Quebec, Canada

**Keywords:** preterm birth, psychiatric disorders, administrative data, effect modification

## Abstract

**Objectives:**

The objectives of this study were to quantify the associations between preterm birth and adolescent-to-adult psychiatric disorders in the Quebec (Canada) population and to determine whether sex and socioeconomic status (SES) modified this relationship.

**Methods:**

This was an observational cohort study using administrative data from the province of Quebec, Canada. All eligible children born preterm between 1976 and 1995 were identified (*N* = 100,040) and matched 1:2 with term-born children. Individuals were followed from age 11 years until either incident diagnosis of a psychiatric disorder (attention-deficit/hyperactivity disorder [ADHD], psychosis, bipolar disorder, anxiety, or depression), death, or December 2019. Preterm birth was considered as a binary (<37 weeks gestational age) and categorical exposure (extreme, <28; very, 28–31; moderate-to-late, 32–36 weeks gestational age), in addition to continuous gestational age in weeks. Cox proportional hazard models were applied. Effect-modifying roles of sex and SES were investigated in interaction analyses.

**Results:**

Compared to term-born children, those born preterm had a higher risk of all outcomes, with magnitudes ranging from HR 1.16 for ADHD (95% confidence interval 1.13, 1.19) to 1.05 for anxiety (1.04, 1.07). A dose-response relationship was observed, with increasing risks of ADHD, psychosis, and anxiety as the degree of preterm birth increased. Despite some statistically significant associations, there was no clinically significant evidence of effect modification by sex or SES.

**Conclusions:**

Children born preterm had an increased risk of psychiatric disorders in adolescence-to-adulthood, with similar risks across sexes and socioeconomic strata of the population. Policies for early and continued mental health surveillance in this susceptible group are important to initiate appropriate interventions.

## Résumé

**Objectifs:**

Cette étude visait à quantifier les liens entre la naissance prématurée et les troubles psychiatriques présentés par une population d’adolescents et d’adultes du Québec (Canada) et à déterminer si le sexe et le statut socio-économique (SSÉ) influaient sur ces liens.

**Méthodologie:**

Il s’agissait d’une étude de cohorte observationnelle utilisant des données administratives provenant de la province de Québec, au Canada. Tous les enfants éligibles nés avant terme entre 1976 et 1995 ont été identifiés (*N* = 100 040) et appariés dans un rapport de 1:2 avec des enfants nés à terme. Les personnes ont fait l’objet d’un suivi à partir de l’âge de 11 ans jusqu’au diagnostic d’un trouble psychiatrique (trouble déficitaire de l’attention/hyperactivité [TDAH], psychose, trouble bipolaire, anxiété ou dépression), jusqu’à leur décès ou jusqu’en décembre 2019. La naissance prématurée a été considérée comme variable binaire (âge gestationnel inférieur à 37 semaines) et catégorique (très grande prématurité, <28; grande prématurité, 28–31; prématurité moyenne à tardive, de 32 à 36 semaines d’âge gestationnel), et l’âge gestationnel a également été considéré comme variable continue. Des analyses selon les modèles de régression de Cox (modèles des risques proportionnels) ont été réalisées. Le rôle du sexe et du SSÉ dans la modification de l’effet a été étudié dans le cadre d’analyses d’interaction.

**Résultats:**

Comparativement aux enfants nés à terme, les prématurés présentaient un risque accru pour tous les résultats, avec des amplitudes allant d’un rapport de risque (RR) de 1,16 pour le TDAH (intervalle de confiance [IC] à 95% : 1,13 à 1,19) à 1,05 pour l’anxiété (1,04 à 1,07). Une relation dose-réponse a été observée, avec une augmentation des risques de TDAH, de psychose et d’anxiété à mesure que le degré de prématurité augmentait. Bien que quelques associations soient statistiquement significatives, il n’y avait aucune preuve cliniquement significative d’une modification de l’effet en fonction du sexe ou du SSÉ.

**Conclusions:**

Les enfants prématurés présentaient un risque accru de troubles psychiatriques à l’adolescence et à l’âge adulte, avec des risques similaires pour les deux sexes et toutes les couches socio-économiques de la population. Il est important de mettre en place des politiques de surveillance précoce et continue de la santé mentale chez ce groupe vulnérable afin de proposer des interventions appropriées.

## Introduction

Preterm birth,^1^ defined as <37 weeks of gestational age (GA), occurs in 8% of Canadian children,^2^ with over 95% surviving into adulthood.^3^ These children experience a greater prevalence of long-term health concerns, including cardiovascular and metabolic issues.^3,4^ Concerns extend to mental health issues, such as attention-deficit/hyperactivity disorder (ADHD),^5–10^ psychosis,^6,7,11–13^ bipolar disorder,^6,11,12^ anxiety,^5,7,9^ and depression.^11,12,14^

Several gaps remain in our understanding of the associations between preterm birth and psychiatric outcomes. Firstly, most studies are based on administrative data from Europe, particularly Scandinavian countries.^7–9,11–15^ As Anderson's theory of health-care utilization states, factors like health care and population structures affect residents’ care-seeking practice.^16–20^ Due to sociodemographic and health-care differences between Europe and North America, triangulating findings from different countries is needed to strengthen evidence for the association between preterm birth and mental health.^3,16^ Secondly, identifying subgroups of preterm individuals at higher risk for psychiatric disorders can help target those who would benefit most from surveillance and intervention programs aimed at improving functioning and quality of life. To this end, studies examining sex differences in the association between preterm birth and psychiatric outcomes have either found higher odds of depression in females but not males,^14^ or no interactions at all.^5,15^ Similarly, one study found a higher risk for psychiatric hospitalization in preterm birth among children from lower socioeconomic status (SES),^15^ while another found no interaction between very preterm/very low birth weight and SES for neurocognitive outcomes in adulthood.^21^

To fill these knowledge gaps, this study investigated the associations between preterm birth and adolescent-to-adult psychiatric disorders using a large population-based administrative database from the Canadian province of Quebec. We additionally investigated the roles of sex and SES as effect modifiers.

## Methods

### Study Design and Population

This cohort study uses a Quebec administrative database linking multiple registers curated by the *Institut de la statistique du Québec:* (1) the Registry of demographic events (RED), (2) the Régie de l'assurance maladie du Québec (RAMQ), including Medical Services, and (3) the Maintenance and data processing for the study of the hospital clientele (Med-Echo) databases. The RED contains information on birthdate, location of birth, sex, GA, birth weight, parental age, maternal education, and child death date. The RAMQ and Med-Echo databases track outpatient medical visits, available to all Quebec residents, and inpatient stays; this includes visit/discharge dates and corresponding medical diagnoses since 1987.

All children born preterm in Quebec between 1976 and 1995 were identified from the RED (*N* = 110,470) and matched 1:2 with term-born children (i.e., 37–42 weeks’ GA) on year of birth, sex, and pregnancy type (single vs. twin) (*N* = 210,400), resulting in a total of 320,870 individuals (Supplementary Figure 1). Preterm and term-born children were either singletons or twins, born between 23 and 42 weeks, inclusive. Given the lack of diagnostic data before 1987 (meaning the earliest cohort members had no diagnostic data before age 11) and the average age of onset for our outcomes being in the age range 5–20 (except ADHD, which can start as early as ages 3–5),^22^ follow-up for mental disorder diagnoses began at age 11. Individuals were followed until 31 December 2019, so the youngest and oldest cohort members would have been 24 and 43 years old, respectively. We excluded those with no maternal data, so the final cohort included 303,375 participants. Compared to the analytic sample, those excluded had a higher proportion of being born extremely preterm (<28 weeks’ GA), were predominantly born in 1976–1981, and had a higher proportion of mothers who were single/never married (Supplementary Table 1).

### Preterm Birth

Preterm birth was defined as GA <37 weeks (vs. term birth 37–42 weeks). Preterm birth was further disaggregated into extreme (<28 weeks), very (28–31 weeks and 6 days), and moderate-to-late (32–36 weeks and 6 days) preterm groups. Lastly, GA was considered as a continuous variable (range: 23–42 weeks).

### Mental Health Outcomes

We considered five primary outcomes that constitute the most common psychiatric disorders: ADHD, psychosis, bipolar disorder, anxiety, and depression. These were identified using codes from the *International Classification of Diseases, Ninth and Tenth Revisions* from outpatient (i.e., physician billing, extracted from the RAMQ Medical Services database, principal diagnosis per visit recorded) and inpatient (i.e., hospital discharge diagnoses, extracted from the Med-Echo services database, principal diagnosis and up to 40 secondary diagnoses per visit recorded) data (Supplementary Table 2). The algorithms used to define these outcomes align with previous studies on mental disorder prevalence in Quebec.^23–26^

### Confounding Factors and Effect Modifiers

Confounding factors, selected based on past literature, included: child birth year (1976–1980, 1981–1985, 1986–1990, 1991–1996); sex (female, male); maternal parity (primiparous, multiparous); birth plurality (single, twin); maternal and paternal age at delivery (continuous – in years); maternal birthplace (Quebec, rest of Canada, other); maternal mother tongue (French, English, other); rural residence (urban, rural); maternal marital status (single/never married, coupled/married, divorced/widowed/separated); and maternal education (continuous – in years). Due to the lack of race and ethnicity data in the administrative database, maternal birthplace and mother tongue were included as covariates instead. SES was assessed using area-based material and social deprivation indices derived from Canadian census data collected every 5 years (1991–2016), according to enumeration area in 1991 and 1996 and dissemination area from 2001 onwards (encompassing 400–700 residents).^27^ Specifically, these were calculated based on those aged ≥15 years with the following indicators: (1) proportion without a secondary studies certificate/diploma; (2) proportion employed; (3) average personal income; (4) proportion living alone; (5) proportion separated, divorced, or widowed; and (6) proportion of single-parent families.^28^ Maternal education was also included as an individual-level SES factor.

### Data Analysis

For our first aim, Cox proportional hazard models were applied to estimate hazard ratios (HRs) and 95% confidence intervals for the associations of preterm birth with each outcome. Individuals were followed from age 11 until the first incidence of the outcome of interest, death, emigration, or end of follow-up (31 December 2019), whichever came first. A first diagnosis of one outcome did not censor participants from follow-up for other outcomes. Three models were fitted: unadjusted (model 1); minimally adjusted, i.e., adjusting for key confounders, namely child sex, maternal education, and material and social deprivation indices (model 2); and fully adjusted, i.e., further adjusted for the remaining confounders (model 3). The robust sandwich estimator accounted for multiple births clustered at mothers. We handled missing confounder values using multivariate imputations by chained equations: models were estimated across 15 imputed datasets, and their estimates were then pooled. We assessed the assumption of proportional hazards by plotting cumulative incidence and using the Schoenfeld test.^29^ For our second aim, we tested for effect modification by sex, maternal education level, social deprivation index, and material deprivation index, applying the minimally adjusted model and using GA as a continuous variable.

We conducted several sensitivity analyses to test the robustness of our results. Firstly, we re-estimated models restricting our sample to those born after 1987 (when diagnostic information became available). We then re-estimated models after removing outliers, defined as GA-birth weight pairings more than 3 standard deviations from the mean. Additionally, we tested whether starting ADHD follow-up at age 3 would alter results, as this disorder is commonly diagnosed in early childhood, by fixing follow-up starting at age 3 years and restricting the sample to those born after 1984 (3 years before diagnostic information became available). Lastly, since mortality risk is higher for children born preterm, we considered mortality as a competing risk for our events of interest using Fine-Gray models to examine the robustness of the primary analysis.^30^

All analyses were performed in R version 4.3.1. This study was approved by the McGill University institutional review board (#A11-M62-23B), and its larger umbrella project was approved by the St. Justine Hospital institutional review board (#2022-3447).

## Results

### Population Characteristics

Of the included cohort (*N* = 303,375), 46% were female. Characteristics by preterm birth status are reported in Table 1, with more detailed characteristics by preterm birth category (extremely, very, and moderate-to-late) in Supplementary Table 3. Participants were followed for 5,161,915–6,721,222 person-years. Psychiatric disorder rates were 2770 cases per 100,000 person-years in the preterm group versus 2562 cases per 100,000 person-years in the term group. Incidence rates by preterm birth category are presented in Table 2.

**Table 1.** Demographic and Socioeconomic Characteristics of Subjects in Study Sample (*N* = 303,375) According to Their Preterm Birth Status.

	Preterm	Term	Overall
	(*N* = 100,040)	(*N* = 203,340)	(*N* = 303,375)
	*N* (%)	*N* (%)	*N* (%)
Sex			
Female	45,990 (45.97%)	92,870 (45.67%)	138,860 (45.77%)
Male	54,050 (54.03%)	110,465 (54.33%)	164,515 (54.23%)
Birth weight			
Mean (SD)	2416 (±627.9)	3324 (±512.0)	3025 (±698.3)
Missing	1023 (1.0%)	1626 (0.8%)	2649 (0.9%)
Year of birth			
1976–1980	22,190 (22.18%)	48,455 (23.83%)	70,645 (23.29%)
1981–1985	22,990 (22.98%)	47,605 (23.41%)	70,595 (23.27%)
1986–1990	25,800 (25.79%)	51,025 (25.09%)	76,825 (25.32%)
1991–1995	29,055 (29.04%)	56,255 (27.67%)	85,310 (28.12%)
Type of pregnancy			
Single	86,405 (86.37%)	184,310 (90.64%)	270,715 (89.23%)
Twins	13,630 (13.63%)	18,525 (9.11%)	32,155 (10.60%)
Missing	0 (0%)	503 (0.2%)	503 (0.2%)
Maternal age at delivery			
Mean (SD)	27.11 (±5.029)	27.21 (±4.702)	27.18 (±4.813)
Missing	11 (0.0%)	12 (0.0%)	23 (0.0%)
Maternal years of education			
Mean (SD)	12.15 (±2.868)	12.44 (±2.898)	12.34 (±2.891)
Missing	5976 (6.0%)	7799 (3.8%)	13,775 (4.5%)
Maternal birthplace			
Québec	56,270 (56.25%)	118,470 (58.26%)	174,740 (57.60%)
Rest of Canada	2525 (2.53%)	4520 (2.22%)	7045 (2.32%)
Outside of Canada	40,035 (40.02%)	78,155 (38.44%)	118,190 (38.96%)
Missing	1207 (1.2%)	2191 (1.1%)	3398 (1.1%)
Material deprivation index			
1 (least deprived)	13,730 (13.72%)	30,815 (15.15%)	44,540 (14.68%)
2	16,370 (16.36%)	34,890 (17.16%)	51,260 (16.90%)
3	16,945 (16.94%)	34,775 (17.10%)	51,720 (17.05%)
4	17,830 (17.82%)	35,290 (17.35%)	53,115 (17.51%)
5 (most deprived)	18,535 (18.53%)	35,875 (17.64%)	54,410 (17.93%)
Missing	16,630 (16.6%)	31,697 (15.6%)	48,327 (15.9%)
Social deprivation index			
1 (least deprived)	17,895 (17.89%)	39,075 (19.22%)	56,970 (18.78%)
2	16,135 (16.13%)	35,060 (17.24%)	51,195 (16.88%)
3	16,560 (16.55%)	35,235 (17.33%)	51,795 (17.07%)
4	16,340 (16.33%)	32,180 (15.83%)	48,515 (15.99%)
5 (most deprived)	16,480 (16.47%)	30,090 (14.80%)	46,570 (15.35%)
Missing	16,630 (16.6%)	31,697 (15.6%)	48,327 (15.9%)
Partner status of mother			
Never married	15,265 (15.26%)	23,860 (11.73%)	39,125 (12.90%)
Coupled	68,365 (68.34%)	147,770 (72.67%)	216,135 (71.24%)
Widowed/separated	2310 (2.31%)	3835 (1.89%)	6145 (2.03%)
Missing	14,097 (14.1%)	27,876 (13.7%)	41,973 (13.8%)
Residence			
Rural	24,745 (24.74%)	52,240 (25.69%)	76,985 (25.38%)
Urban	62,380 (62.36%)	122,485 (60.24%)	184,870 (60.94%)
Missing	12,910 (12.9%)	28,610 (14.1%)	41,520 (13.7%)
Parity of mother			
Primiparous	55,130 (55.11%)	119,445 (58.74%)	174,580 (57.55%)
Multiparous	44,905 (44.89%)	83,890 (41.26%)	128,800 (42.45%)
ADHD			
No	91,310 (91.27%)	188,350 (92.63%)	279,660 (92.18%)
Yes	8730 (8.73%)	14,985 (7.37%)	23,715 (7.82%)
Psychosis			
No	96,440 (96.40%)	197,135 (96.95%)	293,575 (96.77%)
Yes	3595 (3.60%)	6205 (3.05%)	9800 (3.23%)
Bipolar disorder			
No	95,085 (95.05%)	194,530 (95.67%)	289,615 (95.46%)
Yes	4955 (4.95%)	8810 (4.33%)	13,760 (4.54%)
Anxiety			
No	64,955 (64.93%)	134,920 (66.35%)	199,875 (65.88%)
Yes	35,085 (35.07%)	68,415 (33.65%)	103,500 (34.12%)
Depression			
No	76,290 (76.26%)	157,735 (77.57%)	234,020 (77.14%)
Yes	23,750 (23.74%)	45,605 (22.43%)	69,355 (22.86%)
Any diagnosis			
No	53,880 (53.86%)	113,780 (55.96%)	167,660 (55.27%)
Yes	46,160 (46.14%)	89,555 (44.04%)	135,715 (44.73%)

**Table 2.** Incidence Rates of Outcomes According to Gestational Age Category. Incidence Rates Are per 100,000 Person-Years.

	Extremely preterm		Very preterm		Moderately preterm		All preterm		Term		All participants	
	*N*	Incidence rate	*N*	Incidence rate	*N*	Incidence rate	*N*	Incidence rate	*N*	Incidence rate	*N*	Incidence rate
ADHD	250	654.03	865	495.18	7615	398.09	8730	410.68	14,985	339.42	23,715	362.58
Psychosis	110	265.97	360	197.22	3130	158.72	3595	163.90	6205	137.08	9800	145.84
BPD	110	271.17	455	250.74	4390	224.09	4955	227.18	8810	195.82	103,500	1838.15
Anxiety	740	2209.34	3065	2028.77	31,285	1900.04	35,085	1916.30	68,415	1800.49	13,760	206.05
Depression	465	1262.91	2090	1277.89	21,195	1191.75	23,750	1200.19	45,605	1111.52	69,355	1140.37
Any diagnosis	990	3340.38	4065	3002.56	41,105	2737.90	46,160	2770.10	89,555	2561.98	135,715	2629.16

### Association Between Preterm Birth and Diagnosis of Psychiatric Disorders

**Figure 1.**
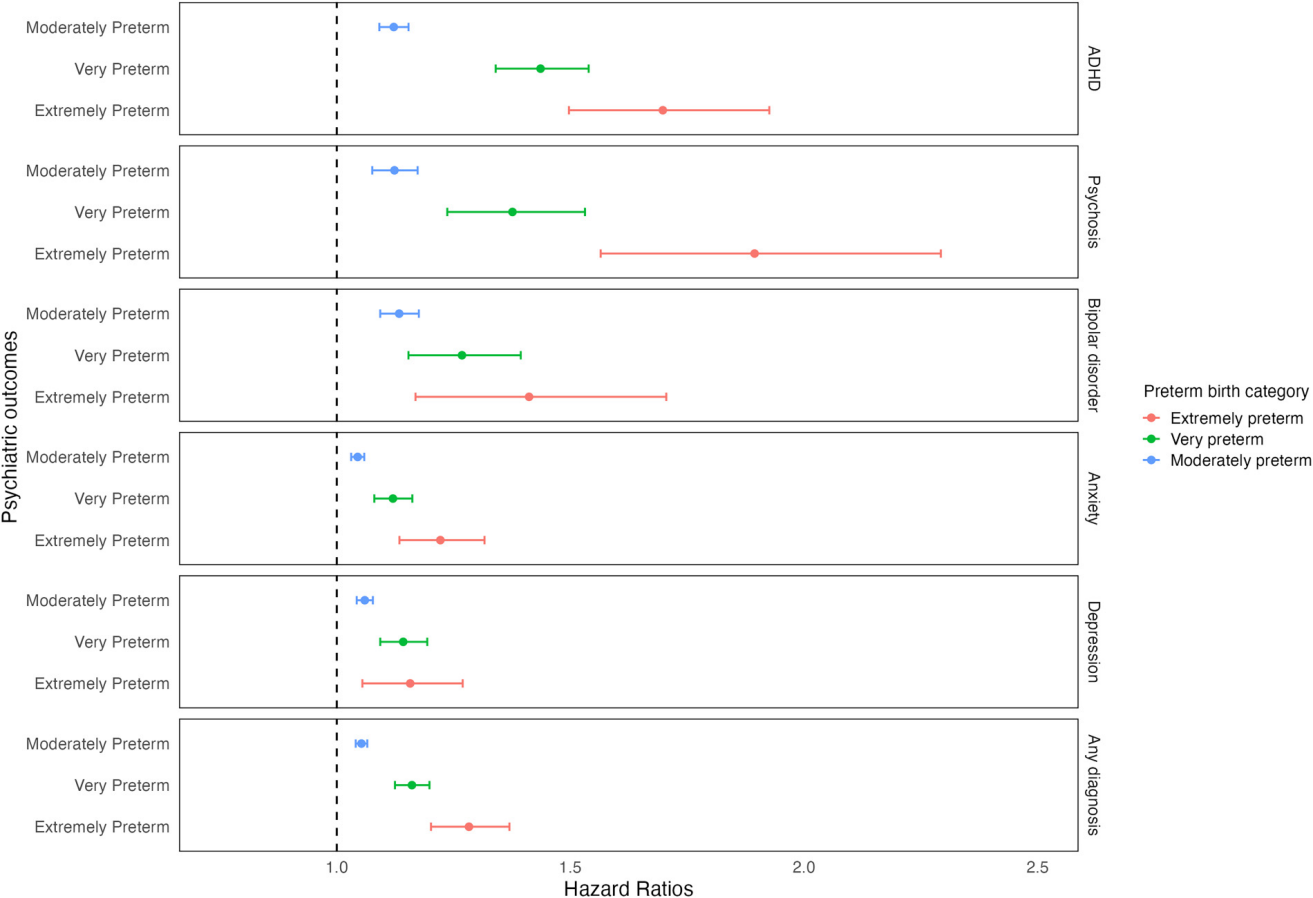
Hazard ratios of the associations between categorical preterm birth and outcomes, using a fully adjusted model with multiply imputed data.

In Cox proportional hazards models, preterm birth was associated with a higher risk of psychiatric disorders, namely ADHD, psychosis, bipolar disorder, anxiety, and depression (Table 3). Risk decreased by an average of 1.5% for each additional gestational week. The strongest association with preterm birth was found for ADHD (HR 1.20, 95% CI 1.17, 1.24), and the weakest for anxiety (HR 1.07, CI 1.05, 1.08). Results remained similar after adjusting for confounders (HR 1.16, CI 1.13, 1.19, and HR 1.05, CI 1.04, 1.07, respectively). When preterm birth categories were considered, HRs increased with degree of preterm birth. For example, the adjusted HRs of psychosis for extremely, very, and moderate-to-late preterm birth were 1.89 (1.56, 2.29), 1.38 (1.24, 1.53), and 1.12 (1.08, 1.17), respectively. This dose-response relationship was clearest for ADHD but not observed for depression (Figure 1). When GA was considered as a continuous variable, each additional gestational week was a protective factor for all outcomes with similar magnitudes across outcomes (e.g., adjusted HR for ADHD, 0.967, CI 0.963, 0.972; adjusted HR for depression, 0.987, CI 0.984, 0.989).

**Table 3.** Associations between Preterm Birth (Binary, Categorical) and Continuous Gestational Age with Outcomes, Using Pooled Results from 15 Imputed Datasets.

	Hazard ratios (95% confidence interval)		
Psychiatric outcomes	Simple model	Minimally adjusted^a^ model	Fully adjusted^b^ model
Model with binary exposure			
ADHD	1.20 (1.17, 1.24)	1.21 (1.18, 1.24)	1.16 (1.13, 1.19)
Psychosis	1.20 (1.15, 1.25)	1.17 (1.13, 1.22)	1.16 (1.11, 1.21)
Bipolar disorder	1.16 (1.12, 1.20)	1.15 (1.11, 1.19)	1.15 (1.11, 1.19)
Anxiety	1.07 (1.05, 1.08)	1.06 (1.04, 1.07)	1.05 (1.04, 1.07)
Depression	1.09 (1.07, 1.1)	1.07 (1.05, 1.09)	1.07 (1.05, 1.09)
Any diagnosis	1.08 (1.07, 1.09)	1.07 (1.06, 1.08)	1.07 (1.05, 1.08)
Model with categorical exposure			
ADHD			
Extremely preterm	1.88 (1.66, 2.13)	1.91 (1.68, 2.16)	1.70 (1.50, 1.93)
Very preterm	1.45 (1.35, 1.55)	1.46 (1.36, 1.56)	1.44 (1.34, 1.54)
Moderately preterm	1.17 (1.14, 1.2)	1.18 (1.14, 1.21)	1.12 (1.09, 1.15)
Psychosis			
Extremely preterm	1.95 (1.62, 2.36)	1.93 (1.60, 2.34)	1.89 (1.56, 2.29)
Very preterm	1.44 (1.29, 1.60)	1.39 (1.25, 1.55)	1.38 (1.24, 1.53)
Moderately preterm	1.16 (1.11, 1.21)	1.14 (1.09, 1.19)	1.12 (1.08, 1.17)
Bipolar disorder			
Extremely preterm	1.41 (1.16, 1.70)	1.37 (1.14, 1.66)	1.41 (1.17, 1.71)
Very preterm	1.28 (1.17, 1.41)	1.26 (1.15, 1.39)	1.27 (1.15, 1.39)
Moderately preterm	1.15 (1.11, 1.19)	1.14 (1.09, 1.18)	1.13 (1.09, 1.18)
Anxiety			
Extremely preterm	1.24 (1.15, 1.34)	1.21 (1.12, 1.30)	1.22 (1.13, 1.32)
Very preterm	1.13 (1.09, 1.17)	1.12 (1.08, 1.16)	1.12 (1.08, 1.16)
Moderately preterm	1.06 (1.04, 1.07)	1.05 (1.03, 1.06)	1.04 (1.03, 1.06)
Depression			
Extremely preterm	1.16 (1.06, 1.27)	1.12 (1.02, 1.23)	1.16 (1.05, 1.27)
Very preterm	1.16 (1.11, 1.21)	1.13 (1.08, 1.18)	1.14 (1.09, 1.19)
Moderately preterm	1.08 (1.06, 1.10)	1.06 (1.05, 1.08)	1.06 (1.04, 1.08)
Any diagnosis			
Extremely preterm	1.31 (1.23, 1.40)	1.28 (1.20, 1.36)	1.28 (1.20, 1.37)
Very preterm	1.17 (1.14, 1.21)	1.16 (1.12, 1.19)	1.16 (1.12, 1.20)
Moderately preterm	1.07 (1.06, 1.08)	1.06 (1.05, 1.07)	1.05 (1.04, 1.07)
Model with continuous exposure			
ADHD	0.960 (0.956, 0.964)	0.959 (0.955, 0.963)	0.967 (0.963, 0.972)
Psychosis	0.963 (0.957, 0.970)	0.967 (0.960, 0.973)	0.968 (0.961, 0.975)
Bipolar disorder	0.976 (0.970, 0.981)	0.977 (0.972, 0.983)	0.976 (0.970, 0.981)
Anxiety	0.988 (0.986, 0.990)	0.990 (0.988, 0.992)	0.989 (0.986, 0.991)
Depression	0.987 (0.984, 0.989)	0.989 (0.986, 0.991)	0.987 (0.984, 0.989)
Any diagnosis	0.985 (0.983, 0.987)	0.986 (0.985, 0.988)	0.986 (0.984, 0.988)

^a^ Minimally adjusted model: adjusted for sex, material deprivation quintile, social deprivation quintile, and maternal education.

^b^ Fully adjusted model: adjusted for sex, material deprivation quintile, social deprivation quintile, maternal education, parity of mother, type of pregnancy, birth period, maternal age, residence, paternal age, maternal birthplace, maternal mother tongue, and maternal partner status.

### Effect Modification by Sex and SES

**Figure 2.**
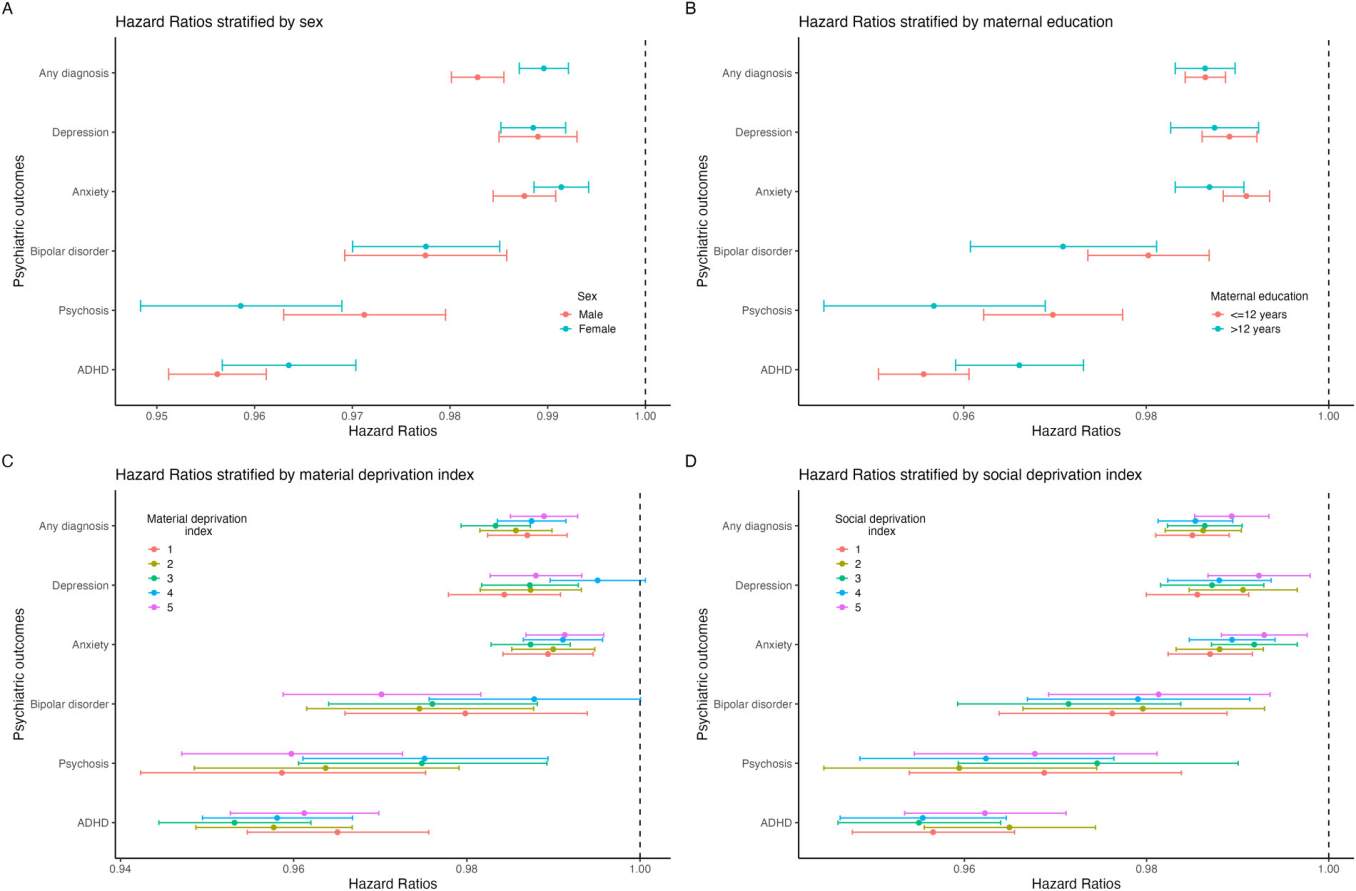
Associations between increasing gestational age and outcomes in samples stratified by (A) Sex, (B) maternal education, (C) material deprivation index, and (D) social deprivation index.

Statistically, effect modification was only seen by sex for ADHD and any diagnosis and by maternal education for psychosis and anxiety (Figure 2, Supplementary Table 4). Interaction models suggested a multiplicative effect in these outcomes, but effect size differences between groups in stratified analyses were minimal. For example, the risk of ADHD in males and females with increasing GA was HR 0.956 (0.951, 0.961) and 0.964 (0.957, 0.970), respectively; the risk of psychosis with increasing GA for those with maternal education >12 years and ≤12 years were 0.957 (0.945, 0.969) and 0.970 (0.962, 0.977), respectively. There was no statistical evidence of effect modification by material and social deprivation quintiles for any outcome.

### Sensitivity Analyses

Sensitivity analyses yielded similar results to the main analysis (Supplementary Table 5).

## Discussion

### Main Findings

Using administrative health data from Quebec, Canada, this population-based cohort study found that preterm birth was associated with increased risk of psychiatric disorders, specifically ADHD, psychosis, bipolar disorder, depression, and anxiety. Dose-response associations were observed, most clearly for ADHD, psychosis, and anxiety, with children of lower GAs more likely to be diagnosed with these psychiatric disorders. Small potential effect modifications by sex were noted for ADHD and any psychiatric diagnosis, and by maternal education for psychosis and anxiety, though given their magnitude, these effects were unlikely to be clinically significant.

### Comparison with Previous Studies

This study is one of the few in North America examining the association between the entire spectrum of preterm birth and mental health outcomes decades into adulthood. Our results align with registry studies from Northern Europe showing a dose-response relationship between preterm birth and adult psychiatric disorders, including ADHD, psychosis, bipolar disorder, and depression.^5,8,10–12^^,15^ On the other hand, an individual participant data meta-analysis of cohort studies reported no consistent increase in self-reported ADHD symptoms in children born preterm,^10^ and another cohort study reported that differences in depression and anxiety between extremely preterm and term groups at 11 years were attenuated at 19 when using clinical cut-offs.^9,31^ These discrepancies may arise from cohort studies capturing milder cases via self-report, whereas register studies using linked hospital data capture more severe cases that require clinical care. Cohorts may also be more likely to have selective attrition by more severe ADHD cases, whereas attrition in register studies is limited by design. On the other hand, if children and adults born preterm are more in contact with the health-care system for other non-mental health-related conditions, this could lead to higher chances of receiving an assessment for psychiatric disorders.^31,32^ Importantly, our results are in line with a Canadian registry study from the province of British Columbia reporting increased risk of anxiety and ADHD in 5- to 15-year-old children born preterm.^33^ However, different from our findings, this study showed no association with depression, likely due to a relatively short follow-up. Therefore, our findings add important information to the literature by showing that the putative effect of prematurity on mental health can emerge only after several decades for some outcomes, like depression.

We found statistical evidence of effect modification by sex or maternal education for select study outcomes. However, differences between sexes were small, thus unlikely to be informative for clinical practice or public health. Although a previous study using a Finnish registry database found extremely preterm birth to be associated with depression only in females,^14^ other registry-based studies have reported no such interaction.^10,15^ Our findings add to this literature, suggesting that while more males are born preterm and preterm-born males are more likely to experience negative physical morbidity into adulthood,^34^ sex differences in psychiatric morbidity are mostly negligible. Similarly, effect modification of SES in our study suggested no differences by area-level SES strata and minimal differences by maternal education for the outcomes of psychosis and anxiety. In contrast, a registry cohort study from Sweden showed that moderately preterm birth is more strongly associated with ADHD medication use in children from families with lower maternal education compared to higher maternal education.^35^ Given the scarcity of available evidence, more studies are needed to clarify whether these differences are country- and/or outcome-specific.

### Interpretations and Implications

Both biological and social mechanisms can explain our associations. Being born preterm interrupts normal development of organ systems, including neurodevelopment. The brain is one of the last organs to develop in utero, with a four-fold increase in cortical volume in the last trimester of gestation.^32^ This growth is disrupted among children born preterm, and studies have detailed the neurological correlates and brain alterations of children and adults born preterm as a consequence,^36–38^ which have been associated with cognitive delay and decreased executive function.^39,40^ This leads to a preterm behavioural phenotype, characterized by inattention, anxiety, and social challenges in childhood,^41^ continuing into diagnosed disorders in adolescence and adulthood.^11,13,42^ With regard to psychosis, it has been suggested that inattention and autism spectrum disorder symptoms earlier in life are prodromic symptoms of psychosis during the transition to adulthood.^39^

The increased risk of mental health problems in preterm birth may also be mediated by psychosocial adversities in the form of early medical interventions and challenges with peer relationships and parenting. Children born extremely preterm are usually admitted to the neonatal intensive care unit for physical interventions immediately following birth. In addition to undergoing medical procedures that could cause pain and distress, infants are separated from their immediate caregivers for the first few days or weeks of life.^32^ This results in stress for both the parents and the child, as attachment during this vulnerable and critical period of life is essential to the parent–child bond. The long-term effects of this may include a disorganized attachment style between the child and their caregivers, particularly in children with longer hospital stays and physical comorbidities, which could in turn contribute to the development of psychopathology in the child.^43,44^ Additionally, children born extremely preterm are more susceptible to bullying,^45,46^ which in itself can contribute to mental health problems.^47^ Increased risk of social adversities is not limited to extremely preterm birth, since bullying has been shown to mediate the relationship between preterm birth with internalizing symptoms in children and with psychotic symptoms in young adults.^46,48^ Additionally, parents with children born preterm are more likely to exhibit stressful behavioural patterns and overprotective parenting styles, which may negatively impact mental health of the child,^44^ highlighting the importance of developing intervention programs that target the entire families.

Our findings have important implications for preventing psychiatric disorders or mitigating the impact of psychiatric symptoms on well-being. Current Canadian guidelines recommend close follow-up until 3 years of age for children born extremely preterm,^49^ but no official guidelines exist about monitoring beyond school age, especially with regard to psychiatric symptoms. Our findings support recommendations from experts in the field that closer follow-up of individuals born preterm is warranted for physical as well as mental health problems.

### Strengths and Limitations

This study is based on a large sample size that includes all preterm births in Quebec over the 20 years between 1976 and 1995, ensuring sufficient statistical power to study rare outcomes and conduct subgroup analyses. Additionally, our follow-up time spanned several decades and continued up to 43 years of age for the oldest subjects, which encompasses the age of onset of the investigated psychiatric disorders and allows us to capture mental health outcomes well into adulthood.

However, the following limitations should be acknowledged. First, our analyses accounted for the main sociodemographic confounding factors, but parental psychiatric history could not be included in our analyses due to the lack of diagnostic data before the year 1987. Additionally, maternal prenatal substance use was not available in the database. While parental psychiatric history is an important confounder, past registry-based studies reported no meaningful impact of familial confounding on the association between preterm birth and psychiatric outcomes. For example, two Swedish registry studies using sibling-control designs found that familial confounding did not account for the dose-response association of preterm birth with ADHD and psychotic/bipolar disorder.^6,50^ Nonetheless, parental psychiatric history is an important predictor of subsequent psychiatric disorder in children born preterm,^51^ and its exclusion could lead to bias in our estimates. The extent of such bias could differ across sex and SES. Indeed, previous studies have shown that parental psychopathology has a different putative impact on male and female children.^56^ Similarly, the effect of parental psychopathology on child mental health may be amplified in low-resource settings.^57^ Therefore, future studies on the association between prematurity and mental health, including parental psychopathology as a confounding factor, should try to replicate our stratified analysis by sex and SES. Similarly, maternal substance use during pregnancy has been causally linked with preterm birth and is associated with mental health consequences in offspring.^52–55^ Two studies from Sweden that studied the association between preterm birth and ADHD medication prescription and diagnosis reported that this association persisted after adjusting for maternal smoking during pregnancy, among other confounders.^35,50^ However, they did not account for other types of substance use. The lack of availability of this information in our database means that the magnitude of our reported associations may be subject to bias. Second, although we included maternal education at the individual level, other socioeconomic indicators used in our study were area-level measures of SES. Thus, residual confounding by SES could not be ruled out in our study.^58^ Third, our outcomes reflect health-care utilization for psychiatric disorders and not necessarily disorder prevalence, since help-seeking behaviour may be affected by several socioeconomic determinants. Furthermore, our database included both principal and secondary diagnoses from hospital discharge documents, but only the principal diagnosis from outpatient physician visits. Therefore, misclassification is possible if a psychiatric disorder was first identified in an outpatient setting but not included as the principal diagnosis for that visit. Fourth, as previously mentioned, the sample excluded because of unavailable outcome data is comprised predominantly of children born very/extremely preterm and in the years 1976–1981 (Supplementary Table 1). Therefore, exclusion was not independent of the exposure, which could have introduced selection bias into our estimates. Finally, subjects were lost to follow-up if they emigrated out of Quebec, as the database does not record emigration or transfer of medical records. Nonetheless, emigration out of Quebec is minimal, around 0.05% as previously reported,^59^ so this is unlikely to substantially impact results.

## Conclusion

Using a large administrative database from Quebec, Canada, this study found that preterm birth was associated with a higher risk of neurodevelopmental, mood, and psychosis-spectrum disorders, with lower GAs associated with increased risk in ADHD, psychosis, and anxiety. These findings support recommendations for enhanced mental health screening and support for children born preterm, especially those born extremely preterm.

## Supplemental Material

sj-docx-1-cpa-10.1177_07067437251389872 - Supplemental material for Preterm Birth and Risk of Psychiatric Disorders: A Register-Linkage Cohort Study: Liens entre la naissance prématurée et le risque de troubles psychiatriques : une étude de cohorte avec couplage de registres Supplemental material, sj-docx-1-cpa-10.1177_07067437251389872 for Preterm Birth and Risk of Psychiatric Disorders: A Register-Linkage Cohort Study: Liens entre la naissance prématurée et le risque de troubles psychiatriques : une étude de cohorte avec couplage de registres by Jude Balit, Ophélie Collet, Seungmi Yang and Sylvana M. Côté, Anne Monique Nuyt, Thuy Mai Luu, Massimiliano Orri in The Canadian Journal of Psychiatry
