# Supplementary material for: Preterm Birth and Risk of Psychiatric Disorders: A Register-Linkage Cohort Study: Liens entre la naissance prématurée et le risque de troubles psychiatriques : une étude de cohorte avec couplage de registres
Source: Can J Psychiatry. 2025 Oct 28;71(2):93–105. doi: 10.1177/07067437251389872 (PMC12568557; doi:10.1177/07067437251389872)
Supplement: sj-docx-1-cpa-10.1177_07067437251389872 - Supplemental material for Preterm Birth and Risk of Psychiatric Disorders: A Register-Linkage Cohort Study: Liens entre la naissance prématurée et le risque de troubles psychiatriques : une étude de cohorte avec couplage de registres [file sj-docx-1-cpa-10.1177_07067437251389872.docx]

**Supplementary tables and figures:**

**Supplementary Figure 1.** Flowchart of analytical sample

All children born preterm in Quebec between 1976 and 1995 (N=110,470) matched 1:2 on month of birth with full-term controls (N=210,400), identified based on birth certificate

N=320,870

Excluded:

Missing maternal information and/or unsuccessful linkage to register databases.

N= 15255

Assign start of follow-up at 11 years

Final cohort members

N=303,375

Excluded:

Censored before/on the start of follow-up because of

- Death before age 11 N=2235

**Supplementary Table 1.** Demographic and socioeconomic characteristics of children (N=318635) according to their inclusion status in primary analyses, after excluding those who died before age 11 years (N=2235).

|  | Included in analytic sample | | | |  |  |
| --- | --- | --- | --- | --- | --- | --- |
|  | No | | Yes | | Overall | |
|  | (N=15255) | | (N=303,375) | | (N=318635) | |
|  | n (%) | | n (%) | | n (%) | |
| **Gestational age, categorical** |  |  |  |  |  |  |
| **Term, 37-42 weeks** | 6360 | (41.67 %) | 203355 | (67.03 %) | 209695 | (65.81 %) |
| **Preterm** |  |  |  |  |  |  |
| 32-36 | 5000 | (56.18 %) | 89770 | (89.73 %) | 94765 | (86.99 %) |
| 28-31 | 1540 | (17.30 %) | 8295 | (8.29 %) | 9835 | (9.03 %) |
| <28 | 2360 | (26.52 %) | 1975 | (1.97 %) | 4335 | (3.98 %) |
| **Gestational age, continuous** |  |  |  |  |  |  |
| Mean (SD) | 34.65 | (± 5.329) | 37.77 | (± 2.840) | 37.62 | (± 3.080) |
| **Sex** |  |  |  |  |  |  |
| Female | 6825 | (44.73 %) | 138860 | (45.77 %) | 145685 | (45.72 %) |
| Male | 8435 | (55.27 %) | 164515 | (54.23 %) | 172950 | (54.28 %) |
| **Birth Weight** |  |  |  |  |  |  |
| Mean (SD) | 2433 | (± 1051) | 3025 |  | 2997 | (± 729.4) |
| Missing | 535 | (3.5%) | 2650 | (0.9%) | 3180 | (1.0%) |
| **Year of birth** |  |  |  |  |  |  |
| 1976-1980 | 7160 | (46.92 %) | 70645 | (23.29 %) | 77805 | (24.42 %) |
| 1981-1985 | 3580 | (23.48 %) | 70595 | (23.27 %) | 74175 | (23.28 %) |
| 1986-1990 | 2495 | (16.35 %) | 76825 | (25.32 %) | 79320 | (24.89 %) |
| 1991-1995 | 2020 | (13.25 %) | 85310 | (28.12 %) | 87335 | (27.41 %) |
| **Type of pregnancy** |  |  |  |  |  |  |
| Single | 13320 | (87.32 %) | 270715 | (89.23 %) | 284040 | (89.14 %) |
| Twins | 1870 | (12.26 %) | 32155 | (10.60 %) | 34025 | (10.68 %) |
| Missing | 65 | (0.43 %) | 505 | (0.17 %) | 570 | (0.18 %) |
| **Maternal age at delivery** |  |  |  |  |  |  |
| Mean (SD) | 26.3 | (± 5.327) | 27.18 | (± 4.813) | 27.13 | (± 4.842) |
| Missing | 5 | (0.0%) | 25 | (0.0%) | 30 | (0.0%) |
| **Maternal years of education** |  |  |  |  |  |  |
| Mean (SD) | 11.73 | (± 3.079) | 12.34 | (± 2.891) | 12.32 | (± 2.903) |
| Missing | 1820 | (11.9%) | 13775 | (4.5%) | 15595 | (4.9%) |
| **Maternal birthplace** |  |  |  |  |  |  |
| Québec | 8525 | (55.88 %) | 174740 | (57.60 %) | 183265 | (57.52 %) |
| Rest of Canada | 1465 | (9.62 %) | 7045 | (2.32 %) | 8515 | (2.67 %) |
| Outside of Canada | 4925 | (32.27 %) | 118190 | (38.96 %) | 123115 | (38.64 %) |
| Missing | 340 | (2.24 %) | 3400 | (1.12 %) | 3740 | (1.17 %) |
| **Material deprivation index** |  |  |  |  |  |  |
| 1 (least deprived) | 230 | (1.52 %) | 44540 | (14.68 %) | 44775 | (14.05 %) |
| 2 | 250 | (1.64 %) | 51260 | (16.90 %) | 51510 | (16.17 %) |
| 3 | 245 | (1.62 %) | 51720 | (17.05 %) | 51965 | (16.31 %) |
| 4 | 285 | (1.87 %) | 53115 | (17.51 %) | 53400 | (16.76 %) |
| 5 (most deprived) | 315 | (2.08 %) | 54410 | (17.93 %) | 54725 | (17.18 %) |
| Missing | 13925 | (91.3%) | 48325 | (15.9%) | 62255 | (19.5%) |
| **Social deprivation index** |  |  |  |  |  |  |
| 1 (least deprived) | 250 | (1.65 %) | 56970 | (18.78 %) | 57220 | (17.96 %) |
| 2 | 215 | (1.41 %) | 51195 | (16.88 %) | 51410 | (16.13 %) |
| 3 | 245 | (1.61 %) | 51795 | (17.07 %) | 52040 | (16.33 %) |
| 4 | 265 | (1.74 %) | 48515 | (15.99 %) | 48780 | (15.31 %) |
| 5 (most deprived) | 355 | (2.32 %) | 46570 | (15.35 %) | 46925 | (14.73 %) |
| Missing | 13925 | (91.3%) | 48325 | (15.9%) | 62255 | (19.5%) |
| **Partner status of mother** |  |  |  |  |  |  |
| Never married | 3470 | (22.76 %) | 39125 | (12.90 %) | 42595 | (13.37 %) |
| Coupled | 9965 | (65.33 %) | 216135 | (71.24 %) | 226105 | (70.96 %) |
| Widowed/Separated | 425 | (2.78 %) | 6145 | (2.03 %) | 6570 | (2.06 %) |
| Missing | 1395 | (9.14 %) | 41975 | (13.84 %) | 43365 | (13.61 %) |
| **Residence** |  |  |  |  |  |  |
| Missing | 5000 | (32.77 %) | 41520 | (13.69 %) | 46520 | (14.60 %) |
| Rural | 2650 | (17.38 %) | 76985 | (25.38 %) | 79640 | (24.99 %) |
| Urban | 7605 | (49.85 %) | 184870 | (60.94 %) | 192475 | (60.41 %) |
| **Parity of mother** |  |  |  |  |  |  |
| Primiparous | 8785 | (57.59 %) | 174580 | (57.55 %) | 183365 | (57.55 %) |
| Multiparous | 6470 | (42.41 %) | 128800 | (42.45 %) | 135270 | (42.45 %) |

**Supplementary Table 2.** Diagnostic codes for mental disorders

|  | International Classification of Diseases 9th Revision | International Classification of Diseases 10th Revision |
| --- | --- | --- |
| ADHD | 314 | F90 |
| Psychosis | 295, 297, 298 | F20, F21, F22, F23, F24, F25, F28, F29 |
| Bipolar disorder | 296 | F30, F31, F34.0 |
| Anxiety | 300.0-3, 300.5-9 | F40, F41, F42, F45 |
| Depression | 300.4, 309.0-2, 311 | F32, F33, F34.1, F41.2, F43.2 |

**Supplementary Table 3.** Comparing subjects according to gestational age, excluding subjects with deaths before 11 years and missing maternal data.

|  | <28 | 28-31 | 32-36 | 37-42 | All |
| --- | --- | --- | --- | --- | --- |
|  | (N=1975) | (N=8295) | (N=89770) | (N=203340) | (N=303375) |
|  | N (%) | N (%) | N (%) | N (%) | N (%) |
| **Sex** |  |  |  |  |  |
| Female | 955 (48.50 %) | 3790 (45.71 %) | 41240 (45.94 %) | 92870 (45.67 %) | 138860 (45.77 %) |
| Male | 1015 (51.50 %) | 4505 (54.29 %) | 48530 (54.06 %) | 110465 (54.33 %) | 164515 (54.23 %) |
| **Birth Weight** |  |  |  |  |  |
| Mean (SD) | 1090 (± 641) | 1640 (± 663) | 2520 (± 534) | 3320 (± 512) | 3020 (± 698) |
| Missing | 39 (2.0%) | 118 (1.4%) | 866 (1.0%) | 1626 (0.8%) | 2649 (0.9%) |
| **Year of birth** |  |  |  |  |  |
| 1976-1980 | 320 (16.22 %) | 1780 (21.47 %) | 20090 (22.38 %) | 48455 (23.83 %) | 70645 (23.29 %) |
| 1981-1985 | 390 (19.77 %) | 2060 (24.81 %) | 20545 (22.88 %) | 47605 (23.41 %) | 70595 (23.27 %) |
| 1986-1990 | 535 (27.22 %) | 2160 (26.05 %) | 23105 (25.74 %) | 51025 (25.09 %) | 76825 (25.32 %) |
| 1991-1995 | 725 (36.80 %) | 2295 (27.68 %) | 26035 (29.00 %) | 56255 (27.67 %) | 85310 (28.12 %) |
| **Type of pregnancy** |  |  |  |  |  |
| Single | 1670 (84.69 %) | 6815 (82.14 %) | 77920 (86.80 %) | 184310 (90.64 %) | 270715 (89.23 %) |
| Twins | 300 (15.31 %) | 1480 (17.86 %) | 11850 (13.20 %) | 18525 (9.11 %) | 32155 (10.60 %) |
| Missing | 0 (0%) | 0 (0%) | 0 (0%) | 503 (0.2%) | 503 (0.2%) |
| **Maternal age at delivery** |  |  |  |  |  |
| Mean (SD) | 27.4 (± 5.41) | 27.0 (± 5.20) | 27.1 (± 5.00) | 27.2 (± 4.70) | 27.2 (± 4.81) |
| Missing | 0 (0%) | 2 (0.0%) | 9 (0.0%) | 12 (0.0%) | 23 (0.0%) |
| **Maternal years of education** |  |  |  |  |  |
| Mean (SD) | 12.3 (± 2.88) | 12.0 (± 2.84) | 12.2 (± 2.87) | 12.4 (± 2.90) | 12.3 (± 2.89) |
| Missing | 193 (9.8%) | 680 (8.2%) | 5103 (5.7%) | 7799 (3.8%) | 13775 (4.5%) |
| **Maternal birthplace** |  |  |  |  |  |
| Québec | 885 (44.75 %) | 4755 (57.34 %) | 50630 (56.40 %) | 118470 (58.26 %) | 174740 (57.60 %) |
| Rest of Canada | 55 (2.79 %) | 205 (2.45 %) | 2270 (2.53 %) | 4520 (2.22 %) | 7045 (2.32 %) |
| Outside of Canada | 990 (50.28 %) | 3220 (38.81 %) | 35825 (39.91 %) | 78155 (38.44 %) | 118190 (38.96 %) |
| Missing | 43 (2.2%) | 116 (1.4%) | 1048 (1.2%) | 2191 (1.1%) | 3398 (1.1%) |
| **Material deprivation index** |  |  |  |  |  |
| 1 | 285 (14.34 %) | 1105 (13.32 %) | 12340 (13.75 %) | 30815 (15.15 %) | 44540 (14.68 %) |
| 2 | 290 (14.75 %) | 1305 (15.72 %) | 14775 (16.46 %) | 34890 (17.16 %) | 51260 (16.90 %) |
| 3 | 315 (15.86 %) | 1440 (17.36 %) | 15190 (16.92 %) | 34775 (17.10 %) | 51720 (17.05 %) |
| 4 | 355 (17.89 %) | 1465 (17.68 %) | 16010 (17.83 %) | 35290 (17.35 %) | 53115 (17.51 %) |
| 5 | 335 (16.88 %) | 1580 (19.07 %) | 16620 (18.52 %) | 35875 (17.64 %) | 54410 (17.93 %) |
| Missing | 400 (20.3%) | 1398 (16.9%) | 14832 (16.5%) | 31697 (15.6%) | 48327 (15.9%) |
| **Social deprivation index** |  |  |  |  |  |
| 1 | 295 (14.85 %) | 1370 (16.54 %) | 16230 (18.08 %) | 39075 (19.22 %) | 56970 (18.78 %) |
| 2 | 295 (14.95 %) | 1305 (15.71 %) | 14540 (16.19 %) | 35060 (17.24 %) | 51195 (16.88 %) |
| 3 | 310 (15.66 %) | 1330 (16.04 %) | 14920 (16.62 %) | 35235 (17.33 %) | 51795 (17.07 %) |
| 4 | 330 (16.73 %) | 1400 (16.90 %) | 14605 (16.27 %) | 32180 (15.83 %) | 48515 (15.99 %) |
| 5 | 345 (17.54 %) | 1490 (17.96 %) | 14645 (16.31 %) | 30090 (14.80 %) | 46570 (15.35 %) |
| Missing | 400 (20.3%) | 1398 (16.9%) | 14832 (16.5%) | 31697 (15.6%) | 48327 (15.9%) |
| **Partner status of mother** |  |  |  |  |  |
| Never married | 295 (14.90 %) | 1415 (17.07 %) | 13555 (15.10 %) | 23860 (11.73 %) | 39125 (12.90 %) |
| Coupled | 1295 (65.53 %) | 5490 (66.15 %) | 61585 (68.60 %) | 147770 (72.67 %) | 216135 (71.24 %) |
| Widowed/Separated | 40 (2.08 %) | 145 (1.76 %) | 2125 (2.36 %) | 3835 (1.89 %) | 6145 (2.03 %) |
| Missing | 345 (17.5%) | 1246 (15.0%) | 12506 (13.9%) | 27876 (13.7%) | 41973 (13.8%) |
| **Residence** |  |  |  |  |  |
| Rural | 435 (22.15 %) | 2065 (24.89 %) | 22245 (24.78 %) | 52240 (25.69 %) | 76985 (25.38 %) |
| Urban | 1350 (68.53 %) | 5210 (62.79 %) | 55820 (62.18 %) | 122485 (60.24 %) | 184870 (60.94 %) |
| Missing | 184 (9.3%) | 1022 (12.3%) | 11704 (13.0%) | 28610 (14.1%) | 41520 (13.7%) |
| **Parity of mother** |  |  |  |  |  |
| Primiparous | 1090 (55.20 %) | 4575 (55.16 %) | 49465 (55.10 %) | 119445 (58.74 %) | 174580 (57.55 %) |
| Multiparous | 885 (44.80 %) | 3720 (44.84 %) | 40305 (44.90 %) | 83890 (41.26 %) | 128800 (42.45 %) |
| **ADHD** |  |  |  |  |  |
| No | 1720 (87.28 %) | 7430 (89.59 %) | 82155 (91.52 %) | 188350 (92.63 %) | 279660 (92.18 %) |
| Yes | 250 (12.72 %) | 865 (10.41 %) | 7615 (8.48 %) | 14985 (7.37 %) | 23715 (7.82 %) |
| **Psychosis** |  |  |  |  |  |
| No | 1865 (94.53 %) | 7935 (95.67 %) | 86640 (96.51 %) | 197135 (96.95 %) | 293575 (96.77 %) |
| Yes | 110 (5.47 %) | 360 (4.33 %) | 3130 (3.49 %) | 6205 (3.05 %) | 9800 (3.23 %) |
| **Anxiety** |  |  |  |  |  |
| No | 1235 (62.60 %) | 5230 (63.07 %) | 58485 (65.15 %) | 134920 (66.35 %) | 199875 (65.88 %) |
| Yes | 740 (37.40 %) | 3065 (36.93 %) | 31285 (34.85 %) | 68415 (33.65 %) | 103500 (34.12 %) |
| **Bipolar disorder** |  |  |  |  |  |
| No | 1865 (94.42 %) | 7840 (94.53 %) | 85380 (95.11 %) | 194530 (95.67 %) | 289615 (95.46 %) |
| Yes | 110 (5.58 %) | 455 (5.47 %) | 4390 (4.89 %) | 8810 (4.33 %) | 13760 (4.54 %) |
| **Depression** |  |  |  |  |  |
| No | 1510 (76.43 %) | 6205 (74.82 %) | 68575 (76.39 %) | 157735 (77.57 %) | 234020 (77.14 %) |
| Yes | 465 (23.57 %) | 2090 (25.18 %) | 21195 (23.61 %) | 45605 (22.43 %) | 69355 (22.86 %) |
| **Any diagnosis** |  |  |  |  |  |
| No | 985 (49.92 %) | 4230 (51.00 %) | 48665 (54.21 %) | 113780 (55.96 %) | 167660 (55.27 %) |
| Yes | 990 (50.08 %) | 4065 (49.00 %) | 41105 (45.79 %) | 89555 (44.04 %) | 135715 (44.73 %) |

**Supplementary Table 4.** Associations between continuous gestational age and outcomes, stratified by sex, maternal education, material deprivation index, and social deprivation index using 1 imputed dataset. All models are minimally adjusted. ^a^

|  | Hazard Ratios (95% CIs) | | | | | |
| --- | --- | --- | --- | --- | --- | --- |
| Stratifying variables | ADHD | Psychosis | Bipolar disorder | Anxiety | Depression | Any diagnosis |
| **Stratified by sex** |  |  |  |  |  |  |
| Male | 0.956 (0.951,0.961) | 0.971 (0.963,0.980) | 0.977 (0.969,0.986) | 0.988 (0.984,0.991) | 0.989 (0.985,0.993) | 0.983 (0.980,0.986) |
| Female | 0.964 (0.957,0.970) | 0.959 (0.948,0.969) | 0.978 (0.970,0.985) | 0.991 (0.989,0.994) | 0.989 (0.985,0.992) | 0.990 (0.987,0.992) |
| **Stratified by maternal education** |  |  |  |  |  |  |
| <=12 years | 0.956 (0.951,0.961) | 0.970 (0.962,0.977) | 0.980 (0.974,0.987) | 0.991 (0.988,0.994) | 0.989 (0.986,0.992) | 0.986 (0.984,0.989) |
| >12 years | 0.966 (0.959,0.973) | 0.957 (0.945,0.969) | 0.971 (0.961,0.981) | 0.987 (0.983,0.991) | 0.987 (0.983,0.992) | 0.986 (0.983,0.990) |
| **Stratified by material deprivation index** |  |  |  |  |  |  |
| 1 (least deprived) | 0.965 (0.955,0.976) | 0.959 (0.942,0.975) | 0.980 (0.966,0.994) | 0.989 (0.984,0.995) | 0.984 (0.978,0.991) | 0.987 (0.982,0.992) |
| 2 | 0.958 (0.949,0.967) | 0.964 (0.949,0.979) | 0.975 (0.962,0.988) | 0.990 (0.985,0.995) | 0.987 (0.982,0.993) | 0.986 (0.982,0.990 |
| 3 | 0.953 (0.944,0.962) | 0.975 (0.961,0.989) | 0.976 (0.964,0.988) | 0.987 (0.983,0.992) | 0.987 (0.982,0.993) | 0.983 (0.979,0.987) |
| 4 | 0.958 (0.949,0.967) | 0.975 (0.961,0.989) | 0.988 (0.976,1.000) | 0.991 (0.987,0.996) | 0.995 (0.990,1.001) | 0.987 (0.984,0.991) |
| 5 (most deprived) | 0.961 (0.953,0.970) | 0.960 (0.947,0.973) | 0.970 (0.959,0.982) | 0.991 (0.987,0.996) | 0.988 (0.983,0.993) | 0.989 (0.985,0.993) |
| **Stratified by social deprivation index** |  |  |  |  |  |  |
| 1 (least deprived) | 0.957 (0.948,0.966) | 0.969 (0.954,0.984) | 0.976 (0.964,0.989) | 0.987 (0.982,0.992) | 0.986 (0.980,0.991) | 0.985 (0.981,0.989) |
| 2 | 0.965 (0.956,0.974) | 0.959 (0.945,0.975) | 0.980 (0.966,0.993) | 0.988 (0.983,0.993) | 0.991 (0.985,0.997) | 0.986 (0.982,0.990) |
| 3 | 0.955 (0.946,0.964) | 0.975 (0.959,0.990) | 0.971 (0.959,0.984) | 0.992 (0.987,0.997) | 0.987 (0.982,0.993) | 0.986 (0.982,0.990) |
| 4 | 0.955 (0.946,0.965) | 0.962 (0.949,0.976) | 0.979 (0.967,0.991) | 0.989 (0.985,0.994) | 0.988 (0.982,0.994) | 0.985 (0.981,0.989) |
| 5 (most deprived) | 0.962 (0.953,0.971) | 0.968 (0.955,0.981) | 0.981 (0.969,0.994) | 0.993 (0.988,0.998) | 0.992 (0.987,0.998) | 0.989 (0.985,0.993) |

^a^ Minimally adjusted model: Adjusted for sex, material deprivation quintile, and social deprivation quintile, and maternal education.

**Supplementary Table 5.** Associations between preterm birth (binary, categorical) and gestational age with outcomes in supplementary analyses using 1 imputed dataset. All models were fully adjusted. ^a^

|  | Hazard Ratios (95% confidence interval) | | | |
| --- | --- | --- | --- | --- |
| Psychiatric outcomes | Sample born >= 1987 | Sample excluding outliers | Fine and Gray model | Sample born >=1984 (ADHD starting age 3) |
| **Model with binary exposure** |  |  |  |  |
| ADHD | 1.16 (1.13,1.19) | 1.16 (1.13,1.19) | 1.16 (1.12,1.19) | 1.19 (1.16,1.22) |
| Psychosis | 1.16 (1.11,1.21) | 1.17 (1.12,1.22) | 1.16 (1.11,1.21) |  |
| BPD | 1.15 (1.11,1.19) | 1.15 (1.11,1.19) | 1.15 (1.11,1.19) |  |
| Anxiety | 1.05 (1.04,1.07) | 1.05 (1.04,1.07) | 1.05 (1.04,1.07) |  |
| Depression | 1.07 (1.05,1.09) | 1.07 (1.05,1.09) | 1.07 (1.05,1.08) |  |
| Any diagnosis | 1.07 (1.05,1.08) | 1.07 (1.05,1.08) | 1.06 (1.05,1.08) |  |
|  |  |  |  |  |
| **Model with categorical exposure** |  |  |  |  |
| ADHD |  |  |  |  |
| - Extremely PTB | 1.7 (1.5,1.93) | 1.71 (1.50,1.94) | 1.68 (1.48,1.91) | 1.93 (1.74,2.15) |
| - Very PTB | 1.43 (1.34,1.54) | 1.43 (1.34,1.54) | 1.43 (1.33,1.53) | 1.51 (1.42,1.60) |
| - Moderately PTB | 1.12 (1.09,1.15) | 1.12 (1.09,1.15) | 1.12 (1.09,1.15) | 1.15 (1.12,1.18) |
| Psychosis |  |  |  |  |
| - Extremely PTB | 1.9 (1.57,2.3) | 2.04 (1.68,2.47) | 1.89 (1.56,2.29) |  |
| - Very PTB | 1.38 (1.24,1.53) | 1.38 (1.24,1.54) | 1.37 (1.23,1.53) |  |
| - Moderately PTB | 1.12 (1.08,1.17) | 1.13 (1.08,1.18) | 1.12 (1.08,1.17) |  |
| BPD |  |  |  |  |
| - Extremely PTB | 1.41 (1.17,1.71) | 1.52 (1.25,1.84) | 1.41 (1.16,1.70) |  |
| - Very PTB | 1.27 (1.15,1.39) | 1.27 (1.15,1.40) | 1.27 (1.15,1.39) |  |
| - Moderately PTB | 1.13 (1.09,1.18) | 1.13 (1.09,1.17) | 1.13 (1.09,1.18) |  |
| Anxiety |  |  |  |  |
| - Extremely PTB | 1.22 (1.14,1.32) | 1.24 (1.15,1.34) | 1.22 (1.13,1.31) |  |
| - Very PTB | 1.12 (1.08,1.16) | 1.12 (1.08,1.16) | 1.12 (1.08,1.16) |  |
| - Moderately PTB | 1.04 (1.03,1.06) | 1.04 (1.03,1.06) | 1.04 (1.03,1.06) |  |
| Depression |  |  |  |  |
| - Extremely PTB | 1.16 (1.06,1.27) | 1.2 (1.09,1.32) | 1.15 (1.05,1.26) |  |
| - Very PTB | 1.14 (1.09,1.19) | 1.14 (1.09,1.2) | 1.14 (1.09,1.19) |  |
| - Moderately PTB | 1.06 (1.04,1.08) | 1.06 (1.04,1.08) | 1.06 (1.04,1.08) |  |
| Any diagnosis |  |  |  |  |
| - Extremely PTB | 1.28 (1.2,1.37) | 1.31 (1.23,1.40) | 1.27 (1.19,1.36) |  |
| - Very PTB | 1.16 (1.12,1.2) | 1.16 (1.13,1.20) | 1.16 (1.12,1.20) |  |
| - Moderately PTB | 1.05 (1.04,1.07) | 1.05 (1.04,1.06) | 1.05 (1.04,1.06) |  |
|  |  |  |  |  |
| **Model with continuous exposure** |  |  |  |  |
| ADHD | 0.968 (0.963,0.972) | 0.968 (0.963,0.972) | 0.97 (0.96,0.97) | 0.962 (0.958,0.965) |
| Psychosis | 0.968 (0.961,0.975) | 0.966 (0.960,0.973) | 0.97 (0.96,0.97) |  |
| BPD | 0.976 (0.970,0.981) | 0.975 (0.969,0.981) | 0.98 (0.97,0.98) |  |
| Anxiety | 0.989 (0.986,0.991) | 0.988 (0.986,0.991) | 0.99 (0.99,0.99) |  |
| Depression | 0.987 (0.984,0.989) | 0.986 (0.984,0.989) | 0.99 (0.98,0.99) |  |
| Any diagnosis | 0.986 (0.984,0.988) | 0.986 (0.984,0.988) | 0.99 (0.98,0.99) |  |

^a^ Fully adjusted model: Adjusted for sex, material deprivation quintile, social deprivation quintile, maternal education, parity of mother, type of pregnancy, birth period, maternal age, residence, paternal age, maternal birthplace, maternal mother tongue, and maternal partner status.
